# A proposed model membrane and test method for microneedle insertion studies

**DOI:** 10.1016/j.ijpharm.2014.05.042

**Published:** 2014-09-10

**Authors:** Eneko Larrañeta, Jessica Moore, Eva M. Vicente-Pérez, Patricia González-Vázquez, Rebecca Lutton, A. David Woolfson, Ryan F. Donnelly

**Affiliations:** Queens University, Belfast School of Pharmacy, 97 Lisburn Road, Belfast BT9 7BL, United Kingdom

**Keywords:** Microneedles, Insertion tests, Polymeric films, Optical coherence tomography

## Abstract

A commercial polymeric film (Parafilm M^®^, a blend of a hydrocarbon wax and a polyolefin) was evaluated as a model membrane for microneedle (MN) insertion studies. Polymeric MN arrays were inserted into Parafilm M^®^ (PF) and also into excised neonatal porcine skin. Parafilm M^®^ was folded before the insertions to closely approximate thickness of the excised skin. Insertion depths were evaluated using optical coherence tomography (OCT) using either a force applied by a Texture Analyser or by a group of human volunteers. The obtained insertion depths were, in general, slightly lower, especially for higher forces, for PF than for skin. However, this difference was not a large, being less than the 10% of the needle length. Therefore, all these data indicate that this model membrane could be a good alternative to biological tissue for MN insertion studies. As an alternative method to OCT, light microscopy was used to evaluate the insertion depths of MN in the model membrane. This provided a rapid, simple method to compare different MN formulations. The use of Parafilm M^®^, in conjunction with a standardised force/time profile applied by a Texture Analyser, could provide the basis for a rapid MN quality control test suitable for in-process use. It could also be used as a comparative test of insertion efficiency between candidate MN formulations.

## Graphical abstract

**Figure:**
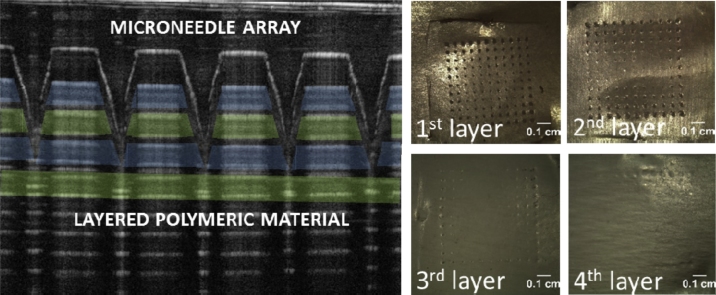

## 1 Introduction

Microneedle (MN) devices are composed of an array of micron-size needles. These systems are currently attracting great interest in transdermal drug delivery research (Chandrasekhar et al., 2013; Henry et al., 1998; Kim et al., 2012; Tuan-Mahmood et al., 2013). MN has the ability to pierce the outermost layer of the skin, the stratum corneum (SC) and create micro-conduits that can deliver drugs to the deeper layers of the skin from where they can be absorbed directly into the systemic circulation (Prausnitz, 2004).

Several key physical factors affect MN performance. These are: type of material, needle height, tip-radius, base diameter, needle geometry and needle density. The penetration depth and the fracture force of MN are determined by all these factors (Davis et al., 2004). Clearly, effective penetration of MN arrays into the skin is the primary pre-requisite for effective drug delivery. However, when developing and testing MN systems, it is apparent that there are limited techniques to evaluate this aspect. Most are based on the measurement of transepidermal water loss (TEWL) (Badran et al., 2009; Bal et al., 2008) or in the visualization of the micropores created after the application of a dye to the skin surface (Oh et al., 2008; Park et al., 2005; Verbaan et al., 2008; Wang et al., 2006). An alternative to these techniques is to take a biopsy of the MN pierced tissue and section it using histological techniques (Badran et al., 2009; Wang et al., 2006; Widera et al., 2006). In this latter case, the subsequent treatment of the skin could change the structure of the micropores. Previously, optical coherence tomography (OCT) has been demonstrated as a good option to evaluate the insertion of MN (Coulman et al., 2011; Donnelly et al., 2010). It is a non-invasive technique and, in addition to pore diameter, the penetration depth of the MN can be readily obtained.

MN insertion studies have typically been performed in biological tissue and this can present some disadvantages, in that tissue samples are often heterogeneous, unstable and difficult to obtain. In addition, the use of biological materials sometimes presents legal issues. Importantly, many of the reported methods, although valuable during the product development phase, are too complex to be suitable as a standard, routine quality control (QC) method for MN. Thus, for QC applications, it is desirable to overcome these limitations by using an artificial material in place of skin. Critically, this will allow the speed and repeatability of experiments to be improved. There are many reports on the use of artificial membranes for drug diffusion studies generally (Ng et al., 2012) and, specifically, for MN mediated transdermal drug delivery (Donnelly et al., 2009; Garland et al., 2012; Zhang et al., 2014). Artificial membranes are widely used for hypodermic needle mechanical testing and bench tests have been developed and standardised for this purpose (Vedrine et al., 2003). However, to the best of our knowledge, studies on artificial membranes for MN insertion or mechanical characterization are scarce (Hamilton, 2011; Koelmans et al., 2013; Muthu, 2007). The implementation of an artificial membrane method for insertion studies can also provide valuable and important comparison tool between different types of MN arrays.

In this work, we propose the use of a polymeric film as a model for MN insertion studies. A comparative study between the insertion of MN into this material and excised neonatal porcine skin was carried out. OCT was used as a tool to evaluate the insertion of MN inside the tissue, taking into account aspects such as the insertion force. Additionally, the force that patients use to apply MN arrays to their skin was evaluated. To the best of our knowledge, there are no studies relating test conditions to actual real life use of MN, in the context of skin insertion by patients, a key factor in designing a reliable QC test method.

## 2 Materials and methods

### 2.1 Materials

Gantrez^®^ S-97 (*M*_w_ = 1.2 × 10^6^), a copolymer obtained from the free acid of methyl vinyl ether and maleic anhydride polymers, was provided by Ashland (Tadworth, Surrey, UK). Poly(ethyleneglycol) (PEG) 10,000 Da was obtained from Sigma–Aldrich (Poole, Dorset, UK). Parafilm M^®^, a flexible thermoplastic sheet (127 μm thickness) made of olefin-type material, was used as skin simulant for insertion studies, was obtained from BRAND GMBH (Wertheim, Germany). Deka^®^ poly(urethane) needle testing foil was provided by Melab GmbH (Leonberg, Germany).

### 2.2 Methods

#### 2.2.1 Preparation of MN arrays

**Fig. 1:**
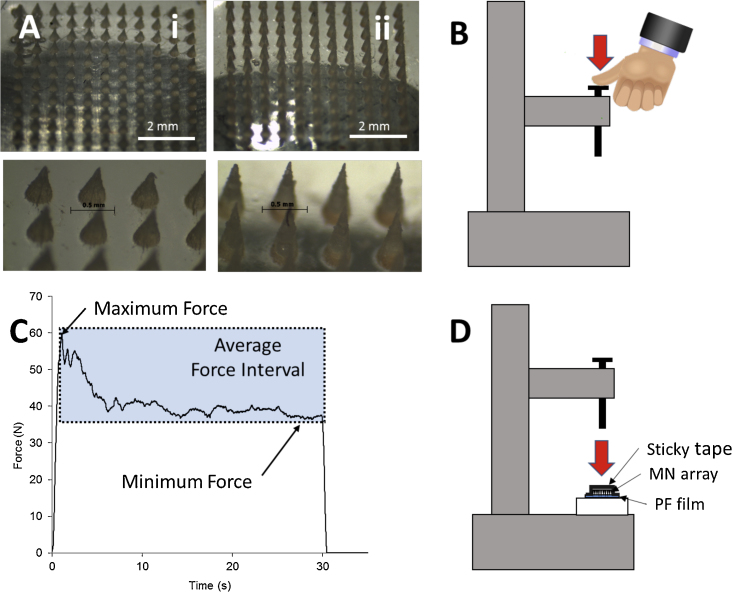
Light microscope images of 600 (i) and 900 (ii) μm length 11 × 11 MN arrays (A). Schematic illustrations: Texture Analyser set up for the measurement of human manual force (B). Example of curve obtained in the manual force measurements (C) and Texture Analyser set up for the insertion of MN arrays (D).

To fabricate MN, aqueous blends containing Gantrez^®^ S-97 (20% w/w) and PEG 10,000 (7.5% w/w) were micromoulded in laser-engineered silicone micromould templates, as previously described (Donnelly et al., 2010, 2011; Migalska et al., 2011; Garland et al., 2011; Singh et al., 2009, 2010). Three different MN geometries were used (Table 1). Light microscope images of the two main MN arrays used in this work can be seen in Fig. 1A. Alternatively, two different formulations were used to prepare either brittle (prepared by adding sodium carbonate 3.5% w/w to the original formulation) or flexible (prepared by replacing PEG in the original formulation with 10% w/w glycerine) MN arrays. In the preparation of brittle and flexible formulations the MN was not crosslinked (Donnelly et al., 2012a).

#### 2.2.2 Human manual force measurements

The forces that 20 volunteers applied using their thumbs were measured using a TA.XTPlus Texture Analyser (Stable Micro Systems, Surrey, UK). The selected volunteers were 10 males and 10 females aged between 20 and 35 years. The volunteers were asked to apply the same force they would use to push an elevator button or to press a stamp onto an envelope, using their right thumb and a 30 s application period, as shown in Fig. 1B. The Texture Analyser was used in tension mode to register the force curves. Three different parameters were determined from these curves: the maximum, minimum and average forces applied during this time interval (Fig. 1C).

#### 2.2.3 Insertion of MN arrays

Full thickness neonatal porcine skin can be considered a good model for human skin in terms of hair sparseness and physical properties (Meyer, 1996). It was obtained from stillborn piglets and excised <24.0 h after birth. Full thickness skin (≈0.5 mm) was then stored in aluminium foil at −20.0 °C until further use. Two sections of skin were placed together, with the dermal side contacting each other, such that the stratum corneum surface was exposed at either side, giving a total skin thickness of about 1 mm. This was then utilised for the OCT assessment of MN penetration.

As an alternative to neonatal porcine skin, Parafilm M^®^ (PF) film and a needle testing polyurethane film were used as skin simulants. A sheet of Parafilm was folded to get an eight-layer film (≈1 mm thickness) and a poly(urethane) needle testing film (Deka^®^) was used as received (0.4 mm thickness). The skin/Parafilm^®^ was then placed onto a sheet of expanded poly(ethylene) for support.

Two insertion methods were carried out: manual and Texture Analyser insertion. For manual insertion, different volunteers were recruited to apply the MN arrays following the same instructions as in the force measurement experiment. The Texture Analyser insertion was performed using a TA.XTPlus Texture Analyser (Stable Micro Systems, Surrey, UK) in compression mode. MN arrays were placed on the surface of the skin/artificial membrane and sticky tape (Office Depot, Boca Raton, USA) was carefully applied on the upper surface without applying force (Fig. 1D). The probe was lowered onto the skin/artificial membrane at a speed of 0.5 mm s^−1^ until the required force was exerted. Forces were held for 30 s and varied from 10 N to 50 N per array. Once the target force was reached, the probe was moved upwards at a speed of 0.5 mm s^−1^.

#### 2.2.4 Optical coherence tomography

Inserted MN arrays were immediately viewed using an EX1301 OCT Microscope (Michelson Diagnostics Ltd., Kent, UK). The swept-source Fourier domain OCT system has a laser centre wavelength of 1305.0 ± 15.0 nm, facilitating real-time high-resolution imaging of the upper skin layers (7.5 μm lateral and 10.0 μm vertical resolution). The skin was scanned at a frame rate of up to 15 B-scans (2D cross-sectional scans) per second (scan width = 2.0 mm). The 2D images were analysed using the imaging software ImageJ^®^ (National Institutes of Health, Bethesda, USA). The scale of the image files obtained was 1.0 pixel = 4.2 μm, thus allowing accurate measurements of the depth of MN penetration and the width of pore created. Three replicates were performed, and the insertion depths of 25 MN were measured in total.

#### 2.2.5 MN insertion testing using light microscopy

MN arrays were inserted using a Texture Analyser, as described above, into eight-layer folded PF sheets. In these cases, sticky tape was not used. After the insertion, the MN arrays were removed from the polymeric sheet. The PF was unfolded and the number of holes in each layer was evaluated using a Leica EZ4 D digital microscope (Leica, Wetzlar, Germany). In order to easily detect the holes created in the PF layers, two polarizer filters were used. The sample was placed between these two filters.

In order to evaluate the thickness of a PF layer, the thickness of 20 samples was evaluated using a digital micrometer (HZH, China). These samples (5) were collected from four different PF rolls.

#### 2.2.6 Statistics

All data are expressed as mean ± standard deviation. Data were compared using a paired, two-tailed Student’s *t*-test. In all cases, *p* < 0.05 was the minimum value considered acceptable for rejection of the null hypothesis.

## 3 Results

### 3.1 Measurement of human volunteers application force

**Fig. 2:**
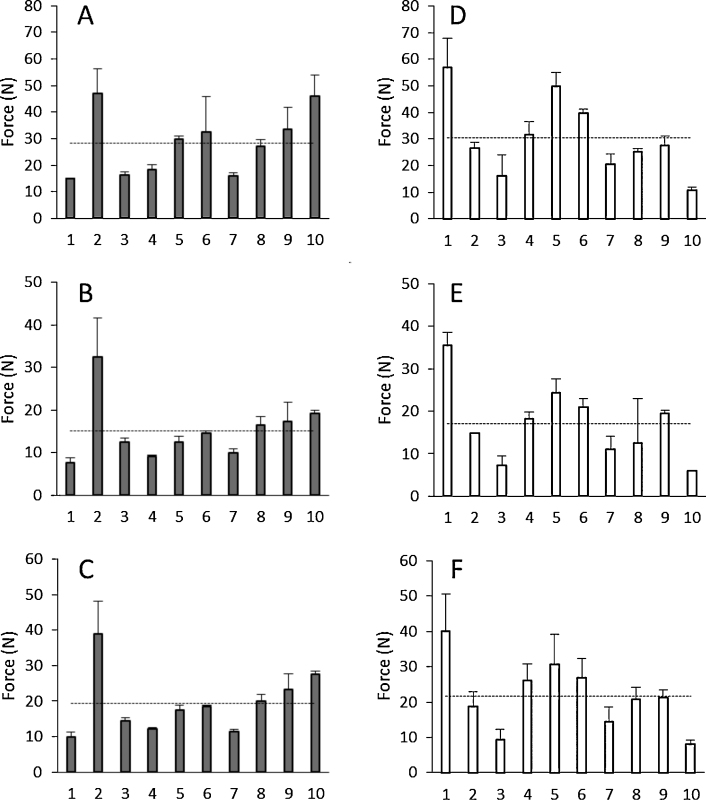
Manual maximum (A and D), minimum (B and E) and average (C and F) forces applied by 10 female volunteers (grey bars) and 10 male volunteers (white bars) in a 30 s time interval. The dashed lines indicate the average force in each case.

Fig. 2 shows the measured force that 20 human volunteers (10 M, 10 F) applied with their thumbs using the same force that they would apply to an elevator button or a postage stamp for 30 s. The obtained average force was around 20 N (19 N F and 21 N M). Additionally, Fig. 2 shows the maximum and minimum forces applied by the volunteers, to illustrate the variability of the applied forces during the 30 s insertion time.

### 3.2 Manual insertion of MN arrays

**Fig. 3:**
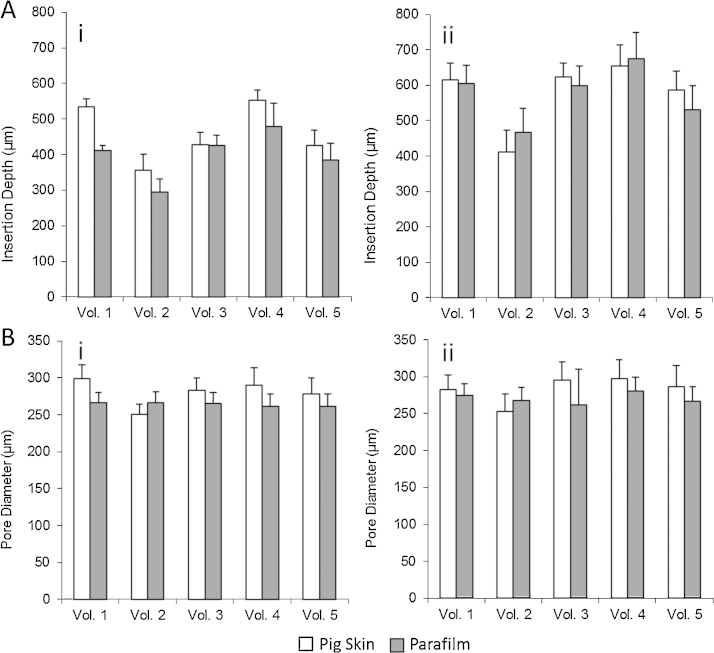
Insertion (A) and pore diameter (B) in pig skin and Parafilm using manual application for 600 (i) and 900 (ii) μm MN arrays.

**Fig. 4:**
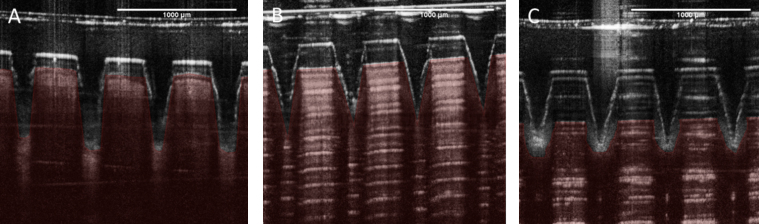
OCT images of a 11 × 11 MN array inserted manually by the same volunteer in different materials: neonatal pig skin (A), eight layers of PF (B) and needle testing film (C). To allow differentiation between MNs a red false colour were applied in the skin/film layers. The original pictures without this colour can be found on the Supplementary content (Fig. S1). (For interpretation of the references to colour in this figure legend, the reader is referred to the web version of this article.)

In order to investigate the influence of the manual application force in the MN insertion and to determine if PF can adequately mimic human skin for insertion studies under the same conditions, a group of five human volunteers were recruited for this study. They were asked to apply a force with their thumbs, as before, to 11 × 11 MN arrays in order to insert them into neonatal porcine skin and PF. Fig. 3A shows the penetration depths of two types of MN arrays evaluated using OCT. There are significant differences between the insertion in skin and in PF in almost all the cases, with PF having lower insertion depths than neonatal porcine skin (*p* < 0.05). Nevertheless, the differences are typically less than the 10% of the total needle length, with the insertion of a 600 μm MN array by volunteer 1 presenting the higher difference at 10.2% of the needle length. However, the range of forces that this volunteer applied was larger than than the rest of the volunteer cohort. Fig. 4A and B shows OCT images of an 11 × 11 MN array inserted into neonatal pig skin and PF respectively, applied by the same volunteer.

In addition, another polymeric membrane was used as skin simulant for insertion studies. This poly(urethane) membrane is normally used for hypodermic needle penetration testing. It was very elastic and, consequently, MN could not penetrate it properly (Fig. 4C). This kind of membrane was previously reported (Koelmans et al., 2013) for MN testing as a stratum corneum simulant with good results. However, the MN in this case were of metal rather than polymeric construction and thus more akin to a hypodermic needle. After insertion of Gantrez polymeric MN, the holes created in this membrane were more like small indentations and were very difficult to see, even using a microscope. In contrast the holes created in PF were easily checked, even without a microscope.

**Fig. 5:**
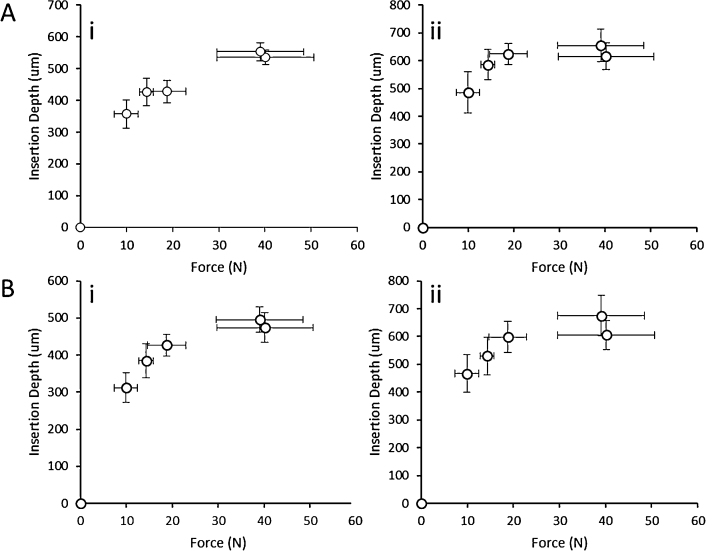
Insertion of 11 × 11 MN in pig skin (A) and PF (B) as a function of the average manual force for different MN heights. 600 (i) and 900 (ii) μm.

The average force that five volunteers applied with their thumbs to the MN arrays during 30 s was measured separately. These results can be correlated with the penetration depths of MN (Fig. 5). As expected, the volunteers that independently were found to apply the highest forces performed the deeper insertions. It is important to notice that in these curves there is a plateau at the end. In addition, as pointed out in the insertion comparison between PF and skin the insertion curves are equivalent. The significant differences can be found for the higher forces. This can be explained considering the higher range of applied forces of these two volunteers. In addition, despite the significant differences between the insertions in both materials (skin and PF), their insertion curves show the same trend and can be overlapped.

### 3.3 Texture Analyser insertion of MN arrays

**Fig. 6:**
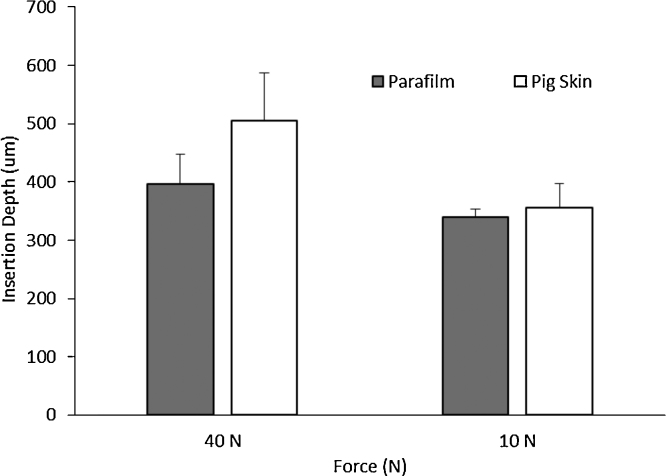
Insertion depths of 600 μm 11 × 11 MN arrays in PF and in neonatal porcine skin for two different forces applied by a Texture Analyser.

In order to use a controlled force for insertion, the Texture Analyser was used. Fig. 6 shows the insertion depths of 11 × 11 MN in PF and in neonatal porcine skin for two different forces applied by a Texture Analyser. As can be seen for the higher force (40 N) there are significant differences between the insertions (*p* < 0.05). This is consistent with the results obtained for manual insertions where the larger differences were present for the higher insertion forces. However, when the insertion force was lower (10 N), significant differences between insertions of MN arrays into neonatal pig skin were not found (*p* = 0.257).

**Fig. 7:**
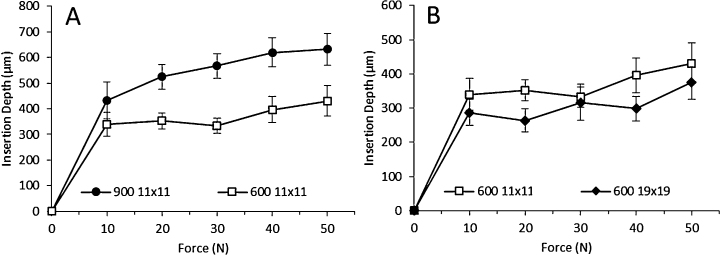
Insertion depths of 11 × 11 (A) and 19 × 19 (B) MN arrays in PF as a function of the force applied by a Texture Analyser.

Fig. 7A shows the insertion depths of 11 × 11 MN in PF as a function of the force applied by a Texture Analyser. As expected, 900 μm MN arrays showed deeper insertions than those of 600 μm (*p* < 0.05). In both cases, the obtained force/insertion curves are similar to those of the same MN arrays inserted manually, reaching the same plateau insertion values (between 300 and 400 μm for 600 μm MN arrays, and between 500 and 600 μm for 900 μm arrays). If the density of the MN in the array is increased the insertion will be different (Verbaan et al., 2008). In Fig. 7B, the comparison between the insertion of 11 × 11 and 19 × 19 MN arrays with the same needle length (600 μm) can be seen. It is noticeable that the penetration depths of 19 × 19 MN arrays are significantly lower than the 11 × 11 arrays (*p* < 0.05). Only for 30 N force can the insertions be considered equivalent (*p* = 0.135), but for these higher forces (30, 40 and 50 N) it is sometimes difficult to ascertain the insertion using OCT for 19 × 19 MN in PF. Thus, the measurements for this MN array (19 × 19 MN) were not very reliable and an alternative method is required.

**Fig. 8:**
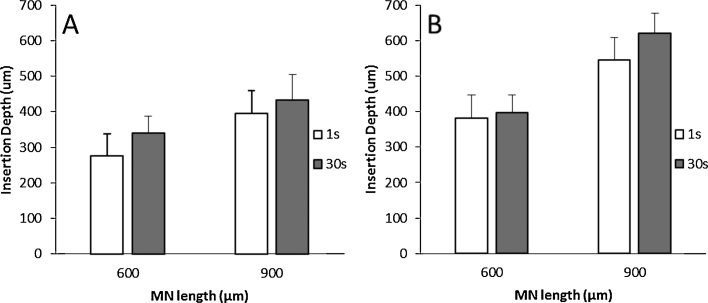
Insertion depths of 600 and 900 μm 11 × 11 MN arrays in PF as a function of the insertion time for two different forces: 10 N (A) and 40 N (B).

All the reported data were obtained by applying certain force with the Texture Analyser during a time period of 30 s. The influence of the application time can be observed in Fig. 8. When the force was applied for only 1 s the insertion was slightly lower. Nevertheless, there are significant differences between insertions of MN arrays during 1 s and 30 s (*p* < 0.05). The only exception is the 40 N insertions of 600 μm MN arrays (*p* = 0.271). This suggests that, for a reliable MN insertion, longer times are preferred. Thus, further tests were carried out using 30 s as the insertion time.

### 3.4 MN insertion testing using light microscopy

**Fig. 9:**
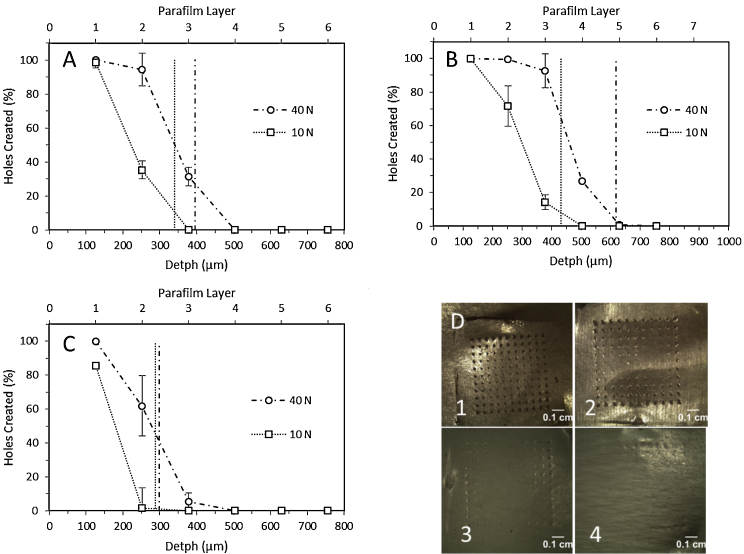
Percentage of holes created in each PF layer using two different insertion forces (40 N and 10 N) for different types of MN arrays: 600 μm 11 × 11 (A), 900 μm 11 × 11 (B) and 600 μm 19 × 19 (C). The dashed lines correspond with the obtained values of insertion depth using OCT in each case (see Fig. 5). Photograph of PF layers after the insertion of 600 μm 11 × 11 MN arrays using a force of 40 N as insertion force: first layer (1), second layer (2), third layer (3) and fourth layer (4).

An alternative to OCT in PF insertion studies could be the evaluation of the number of holes created in each layer of the PF sheet after the application of a MN array (Fig. 9D). Fig. 9 shows the percentage of holes created in each PF layer for different types of MN arrays and two different forces (10 and 40 N). After measuring the average thickness of a PF layer (126 ± 7 μm) the percentage of MN inserted as a function of the depth can be calculated (Fig. 9). As expected using an insertion force of 40 N, 900 μm 11 × 11 MN arrays reached lower PF layers than 600 μm 11 × 11 MN examples. However, 19 × 19 arrays were found to have only pierced two PF layers. These results are consistent with those obtained using OCT. The same insertion profiles were observed with a lower insertion force (10 N) but the MN arrays did not pierce as many layers of PF.

These results can be compared with the insertion depths obtained using OCT (dashed vertical lines in Fig. 9). The number of holes created decreased with the insertion depth. However, it is important to remember that, in the OCT-based study, the factor that is evaluated is whether the needles went through an entire layer of PF. Sometimes it is difficult to determine if the created hole extends across the whole PF layer. Consequently, this test can yield a range of depths of 126 μm instead of an exact value of insertion depth.

**Fig. 10:**
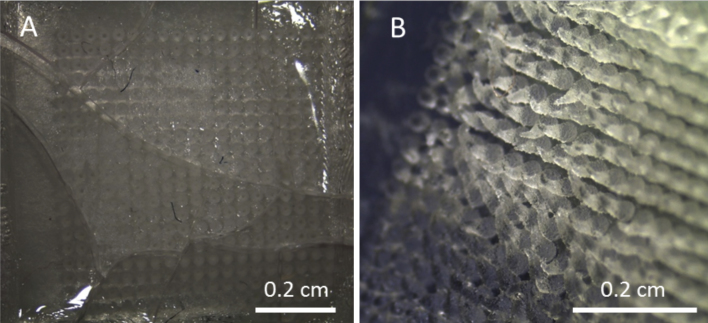
Photograph of 600 μm 19 × 19 MN arrays after insertion in PF of two different formulations: Na_2_CO_3_ formulation (A) and glycerine formulation (B). The insertion force was 40 N in both cases.

Not all MN arrays can be inserted successfully. There are formulations that can fail the insertion test. For a prospective QC test to be useful, it must be able to demonstrate lack of penetration for ineffective formulations. Thus, two formulations were used to prove two different failure cases. The first formulation was that used throughout this work but with an additional 3.5% (w/w) of sodium carbonate that acts as a pore forming agent in order to make the formulation more swellable. These MN were not heated at 80 °C to crosslink the Gantrez polymer. Thus, the resulting MN was very brittle and cannot be properly inserted into the PF because the base plate of the array was broken after application of a 40 N force (Fig. 10A). Replacing the PEG with glycerine and avoiding the crosslinking step resulted in a very flexible formulation. As can be seen in Fig. 10B, these formulations could not been inserted because the MN tips were bent (Fig. 10B).

## 4 Discussion

Scale-up of manufacturing technologies is a key challenge in the development of polymeric MN transdermal systems. An important aspect is the availability of a rapid, simple and standardised QC test method to determine MN penetration characteristics. Such a test is also important in MN array development as a tool to compare candidate MN formulations. Since skin is highly variable and presents a number of other difficulties for these purposes, the identification of an inexpensive, readily obtainable and inexpensive model membrane is important if such a test is to be developed.

In developing a suitable insertion test it is important to know how MN will be used. As an emerging pharmaceutical technology, MN should be patient-friendly, i.e. easy to apply and to remove. In many MN studies, the arrays are applied using various types of applicators (Donnelly et al., 2012b). The use of these devices makes the insertion force very reproducible. However, the use of manual application, if possible, will simplify the insertion process for the patient. In a previous study, manual self-application of MN arrays was evaluated successfully (Donnelly et al., 2014). The main drawback of manual insertion is that the force that an individual applies to a MN array can vary depending on different factors such as age, gender and body weight. Knowing this range of forces could be very useful to design a standardised insertion test for future MN studies.

In this work, we developed a simple experiment to measure the range of forces that patients can apply to polymeric MN arrays. The obtained average forces were about 20 N (Fig. 2). In previous work, the range of forces needed to insert different types of MN was found to be lower than 20 N (Davis et al., 2004; Koelmans et al., 2013; Kong et al., 2011). It is noticeable that the average forces for MN insertion between male and female volunteers was not significant. The volunteer insertion force data inform the range of forces that should be used in a standardised MN insertion test using an artificial membrane.

In order to evaluate if PF is a good alternative to skin for this kind of test, manual insertion in neonatal pig skin and PF were carried out using human volunteers. The results obtained in these experiments suggest that, despite presenting slightly lower penetration depths than porcine skin, PF could be a promising material to replace biological tissue for insertion studies. In previously reported studies, different kinds of artificial films and material were used to test MN (Hamilton, 2011; Koelmans et al., 2013; Muthu, 2007; Zhang et al., 2014). The insertion in these cases was about 60% of the total needle length, but these results correlate well with the insertion depths obtained in an earlier study using the same type of polymeric MN in human volunteers with manual application (Donnelly et al., 2014).

The forces that five volunteers applied with their thumbs can be correlated with MN penetration depths (Fig. 5). In these curves a plateau can be found for higher forces. This means that, due to skin mechanical properties, this kind of MN cannot be totally inserted using a range of forces between 0 and 50 N. Importantly, this behaviour was also seen in PF insertions.

In order to have a more systematic insertion study, a Texture Analyser was used to insert different MN arrays. The first step was to compare the insertion in neonatal porcine skin and PF. The insertion forces were selected taking into account the data obtained from the human volunteer study (range between 0 and 50 N per array). The results showed that there were no significant differences between the two membranes in respect of MN insertion depths, but only for relatively low insertion forces (10 N/array). This is consistent with results obtained for manual insertion of MN arrays, where the lower differences between penetration depths were found for the lower insertion forces.

If PF is going to be used for further studies as a model membrane in MN insertion testing, it is of crucial importance to control and evaluate the behaviour and insertion characteristics when MN are inserted. The insertion depth/force curves (Fig. 6) for different MN arrays presented the same pattern as those obtained with manual insertion, with a plateau for higher forces. This demonstrates that the insertion profiles of these kind of MN arrays are very reproducible and do not depend strongly on how the force is applied.

Another noticeable aspect is that the depths of insertion of 19 × 19 MN arrays are lower than that of 11 × 11. This is consistent with the bed of nails effect (Verbaan et al., 2008). Sometimes, this effect is not observed experimentally depending on the number of MN per array or the insertion method (Verbaan et al., 2008; Widera et al., 2006).

Finally, the last aspect that was evaluated was the insertion time. By reducing this time to 1 s for a defined force value, it was found that the insertion depths in PF are similar (Fig. 7). However, for fully effective insertion, longer application times will improve the penetration depth.

Taking into account all of the data, an easy, inexpensive and reliable insertion test is proposed. The PF membrane used for these insertion studies consisted of eight folded layers. This method allows the evaluation of the number of holes created in each layer of the PF sheet. As the thickness of each layer is known, the insertion depth can be readily estimated without using complex techniques such as OCT, making the test very suitable as an in-process QC method. The insertion profiles obtained (Fig. 8) are consistent with the insertion depths obtained with OCT (Fig. 6). The two different forces that were used in this test have two purposes. The lower force (10 N) will give insertion profiles equivalent to those obtained using neonatal pig skin (Fig. 6) and the higher force (40 N) is used to test the strength of the base plate (Fig. 10). Therefore, this test can be used to discriminate faulty MN arrays.

## 5 Conclusion

The proposed MN insertion test can be easily implemented as a routine method to compare MN formulations and to control the quality of MN arrays. This can be of value in scaled-up MN production processes. The test proposed in this study can be used to complement existing techniques for the physical characterization of MN arrays. The key aspects are the identification of PF as a suitable skin simulant for MN insertion and the development of a facile, rapid and reliable insertion test with potential for use as a QC test method, or for comparative formulation studies.

## Figures and Tables

**Table 1:** Physical properties of the different MN arrays.

MN per array	Height (μm)	Width at base (μm)	Interspacing at base (μm)
11 × 11	600	300	300
11 × 11	900	300	300
19 × 19	600	300	50
