# Supplementary material for: A proposed model membrane and test method for microneedle insertion studies
Source: Int J Pharm. 2014 Sep 10;472(1-2):65–73. doi: 10.1016/j.ijpharm.2014.05.042 (PMC4111867; doi:10.1016/j.ijpharm.2014.05.042)
Supplement: Supplementary file 1 [file mmc1.doc]

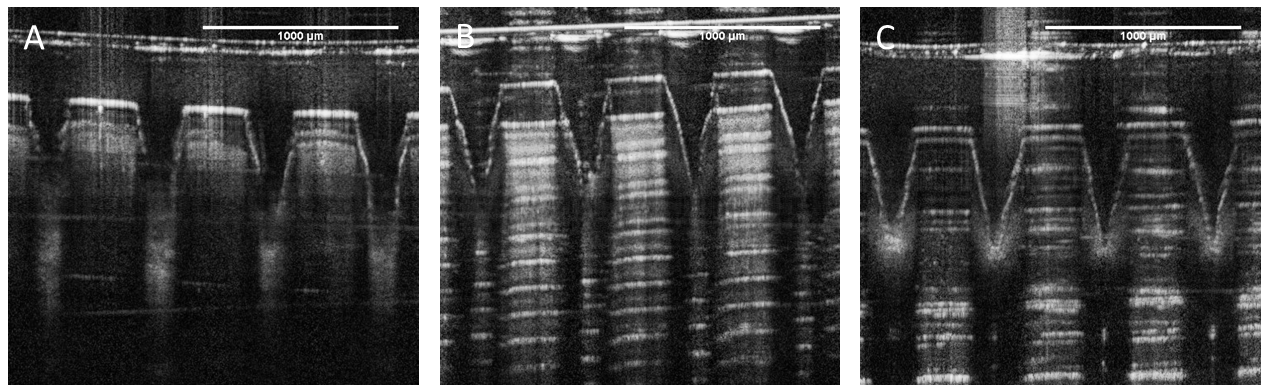


**Figure S1.** OCT images of a 11x11 MN array inserted manually by the same volunteer in different materials: neonatal pig skin (A), 8 layers of PF (B) and needle testing film (C).
